# Antagonistic activity of auxin and cytokinin in shoot and root organs

**DOI:** 10.1002/pld3.121

**Published:** 2019-02-25

**Authors:** Jasmina Kurepa, Timothy E. Shull, Jan A. Smalle

**Affiliations:** ^1^ Department of Plant and Soil Sciences University of Kentucky Lexington Kentucky

**Keywords:** apical meristems, auxin, cytokinin, hormonal antagonism, roots, shoots

## Abstract

The hormones auxin and cytokinin are essential for plant growth and development. Because of the central importance of root and shoot apical meristems in plant growth, auxin/cytokinin interactions have been predominantly analyzed in relation to apical meristem formation and function. In contrast, the auxin/cytokinin interactions during organ growth have remained largely unexplored. Here, we show that a specific interaction between auxin and cytokinin operates in both the root and the shoot where it serves as an additional determinant of plant development. We found that auxin at low concentrations limits the action of cytokinin. An increase in cytokinin level counteracts this inhibitory effect and leads to an inhibition of auxin signaling. At higher concentrations of both hormones, these antagonistic interactions between cytokinin and auxin are absent. Thus, our results reveal a bidirectional and asymmetrical interaction of auxin and cytokinin beyond the bounds of apical meristems. The relation is bidirectional in that both hormones exert inhibitory effects on each other's signaling mechanisms. However, this relation is also asymmetrical because under controlled growth conditions, auxin present in nontreated plants suppresses cytokinin signaling, whereas the reverse is not the case.

## 1 INTRODUCTION

The Arabidopsis cytokinin response pathway is a two‐component signaling mechanism that starts with a family of Arabidopsis histidine kinases receptors that self‐phosphorylate after binding cytokinin. Receptors then transfer the phosphate to a family of Arabidopsis histidine phosphotransfer proteins, which in turn phosphorylate members of two antagonistically functioning families of Arabidopsis Response Regulator proteins (ARRs) (Kieber & Schaller, 2010). The cytokinin response promoting type‐B ARRs are transcription factors expressed in their latent forms and are activated by phosphorylation to induce the expression of primary cytokinin response genes (Argyros et al., 2008; Ishida, Yamashino, Yokoyama, & Mizuno, 2008). The members of the second family of ARRs, the type‐A ARRs, are encoded by primary cytokinin response genes and they repress the primary response by a negative feedback mechanism (To et al., 2004). In contrast to the sequence of activation steps that characterize cytokinin signaling, auxin signaling is based on a repression‐relief mechanism (Lavy & Estelle, 2016). Auxin is perceived by two families of co‐receptors, the SCF^TIR1/AFB^ family of ubiquitin ligases and their targets, the AUX/IAA family of auxin response inhibitors. Auxin acts as a molecular glue that strengthens the interaction of the co‐receptors leading to the degradation of AUX/IAAs by the 26S proteasome and consequent derepression of the transcriptional response activators, the Auxin Response Factors (ARFs) (Roosjen, Paque, & Weijers, 2018). Similar to the cytokinin response pathway, the auxin signaling mechanism also contains a feedback inhibition loop involving the auxin‐induced expression of the response inhibitory AUX/IAA family (Strader & Zhao, 2016).

It is well documented that plant development is to a large extent controlled by interactions between auxin and cytokinin (Schaller, Bishopp, & Kieber, 2015). The existence of this hormonal crosstalk implies that, in addition to negative feedback mechanisms regulating the strength and duration of individual hormonal responses, there are also regulatory mechanisms that calibrate the intensity of a hormone response based on spatial and temporal characteristics of the hormonal interaction. These interactions are of particular importance during the development of shoot and root apical meristems. Auxin and cytokinin were shown to interact both antagonistically and synergistically during the development of root and shoot apical meristems and these interactions involve coordination of signaling, biosynthesis, and transport pathways to delineate stem cell niches, meristem growth, vascular pattern formation, and organ initiation in the shoot (Besnard et al., 2014; Bishopp et al., 2011; Chickarmane, Gordon, Tarr, Heisler, & Meyerowitz, 2012; Dello Ioio et al., 2008; Truskina & Vernoux, 2018). Auxin–cytokinin interactions have also been shown to be essential for the control of the development of determinate meristems such as those needed for gynoecium development (Muller, Larsson, Spichal, & Sundberg, 2017; Reyes‐Olalde et al., 2017).

Although significant progress has been made in understanding the auxin–cytokinin interactions in apical meristem development and function, less is known about the importance and extent of these interactions during the growth of organs after they have been initiated by apical meristems. Significant progress has also been made in understanding the differences between the mechanisms governing the formation of lateral shoot and root organs. In the shoot, leaves are initiated at the shoot apical meristem and these leaf primordia subsequently engage in coordinated cell division, expansion, and differentiation until the final leaf size and shape are reached (Bar & Ori, 2014). Consequently, the shoot apical meristem defines the position of leaves, but their final size and shape are also controlled by signaling mechanisms that reside within the leaves. Lateral organ formation in roots is essentially different in that lateral roots are not formed by the root apical meristem but instead develop from new meristems which derive from pericycle cells. Whether or not pericycle cells engage in the cell division needed to develop lateral root meristems depends on priming by the root apical meristem (Laskowski & Ten Tusscher, 2017) and also on signaling mechanisms that reside outside of the root apical meristem. Thus, both shoot and root growth are modulated by regulatory mechanisms that act beyond those that reside in the apical meristems. Here, we investigate the involvement of auxin–cytokinin interactions at this regulatory level. The current state of knowledge of plantwide reciprocal control of auxin and cytokinin responses is somewhat conflicting. For example, the expression levels of the primary cytokinin‐inducible type‐A *ARR* genes were shown to be downregulated in the auxin‐resistant mutant *axr3‐1*, suggesting that auxin promotes type‐A *ARR* expression (Overvoorde et al., 2005). However, the expression of the same gene set was shown to be downregulated by auxin treatment (Lee, Park, Lee, & Kim, 2009). By using an array of cytokinin and auxin response mutants in combination with transgenic auxin and cytokinin signaling reporter lines, we show here that auxin limits the cytokinin response in both the shoot and root. This one‐directional signaling inhibition is overpowered by cytokinin treatments which lead to auxin response inhibition. Higher concentrations of both hormones convert antagonistic interactions into additive signaling.

## 2 EXPERIMENTAL PROCEDURES

### 2.1 Materials

Reagents were obtained from the following sources: Murashige and Skoog media from Phytotechnology Laboratories (Shawnee Mission, KS); 6‐benzyladenine (BA), 2‐isopentenyladenine (2‐iP) and 1‐naphthaleneacetic acid from Sigma‐Aldrich (St. Louis, MO); 5‐bromo‐4‐chloro‐3‐indolyl‐β‐D‐glucuronic acid (X‐Gluc) from Gold Bio Technology (St. Louis, MO).

### 2.2 Plant lines and plant growth conditions

*Arabidopsis thaliana* plants were grown on 0.8% agar plates with half‐strength Murashige and Skoog medium with 1% sucrose (MS/2, pH 5.7) in a controlled environment chamber at 22°C with a day/night cycle of 16‐hr light (140 μmol photons m^−2^ s^−1^)/8‐hr dark. The Col‐0 ecotype was used as wild‐type control for all experiments and the list of all mutant and transgenic lines used or generated in this study is presented in Supporting Information Table S1. Before introgressing β‐glucuronidase (GUS) reporter genes into various mutant and transgenic backgrounds, the reporter lines *ARR5p:GUS* and *DR5p:GUS* were first backcrossed to the Col‐0 wild type.

For the generation of double and triple mutant and transgene combinations, putative homozygous double and triple mutant and transgenic lines were selected based on their phenotypes; their antibiotic resistances and their genotypes were confirmed by DNA analyses using gene‐specific primers and GUS staining.

The *35Sp:ARR5* transgene in the *Agrobacterium tumefaciens* vector pEarlyGate202 was described earlier (Li, Kurepa, & Smalle, 2013). The *ARR5p:GUS axr3‐3* and *ARR5p:GUS arf7‐1* lines were transformed with the *35Sp:ARR5* transgene by the floral dip method (Clough & Bent, 1998). For the transformation of *ARR5p:GUS axr3‐3*, we found that 5 of 12 transformed lines had larger rosettes that contained less anthocyanin when compared to the untransformed line. These lines also had the strongest reduction in *ARR5p:GUS* expression, an expected effect of the *35Sp:ARR5* transgene. For the transformation of *ARR5p:GUS arf7‐1*, 6 of 13 transformed lines had rosettes larger and with less anthocyanin than the *ARR5p:GUS arf7‐1*. All plants from T1 plants with larger rosettes also had the strongest reduction in *ARR5p:GUS* expression, which is an expected effect of the *35Sp:ARR5* transgene.

### 2.3 Hormone treatments and histological analyses

To test the effects of hormone treatments on the expression of the *ARR5p:GUS* and *DR5p:GUS* transgenes, seedlings were germinated on MS/2 medium and after 4 or 5 days of growth were transferred MS/2 medium with the denoted hormone concentrations and further incubated for 6 hr. GUS activity was assayed by transferring the seedlings to a staining buffer (10 mM Na_2_EDTA, 100 mM NaH_2_PO_4_, 0.1% Triton X‐100) with the X‐Gluc substrate (1 mg/ml). The assays were stopped, and seedlings were cleared by replacing the staining buffer with ethanol and then with a 50% glycerol solution. Different incubation times were used for the GUS activity assays dependent on the aim of the experiment. For all experiments, a minimum of three biological replicates were performed with a minimum of 10 seedlings per treatment. Stained seedlings that were representative of the experimental results were then photographed.

### 2.4 RNA gel blot analysis

Total RNA was prepared using Trizol reagent (Life Technologies, http://www.lifetechnologies.com). The RNA gel blot analyses and the preparation of antisense *ARR5* probe were performed as described (Smalle et al., 2002).

### 2.5 Root inductions from root explants

Roots of plants grown vertically for 6 days were excised and transferred to growth media supplemented with the 1 μM NAA and a number of doses of 2‐iP. A minimum of 15 root explants per line was tested for each NAA/2‐iP concentration combination. Test plates were kept in a controlled environment chamber with continuous light and temperature of 22°C and were followed daily. The root induction data were derived from three biological replicates.

### 2.6 Anthocyanin content

Ten seedlings per sample were submerged into 500 μl of acid methanol (1% HCl) and rocked at 4°C for 12 hr in darkness. The anthocyanin fraction was extracted using chloroform phase separation as described (Kubasek et al., 1992). The anthocyanin content was measured using a DTX 880 Multi‐mode Detector (Beckman Coulter) with a 520/8 nm absorbance filter. All anthocyanin data shown are based on a minimum of three biological replicates.

### 2.7 Lateral root number

Lateral root number was determined by counting by eye all visible lateral roots of any length and developmental stage. All lateral root data shown are based on a minimum of three biological replicates.

### 2.8 Statistical analyses

The descriptive statistics, plotting, and statistical analyses were done using Prism 6 (GraphPad). The statistical tests used to analyze the data, the size of tested sample sets, and number of biological replicates are stated in the Sections 3 and 4 or Figure legends.

## 3 RESULTS

### 3.1 Auxin inhibits cytokinin signaling

**Figure 1:**
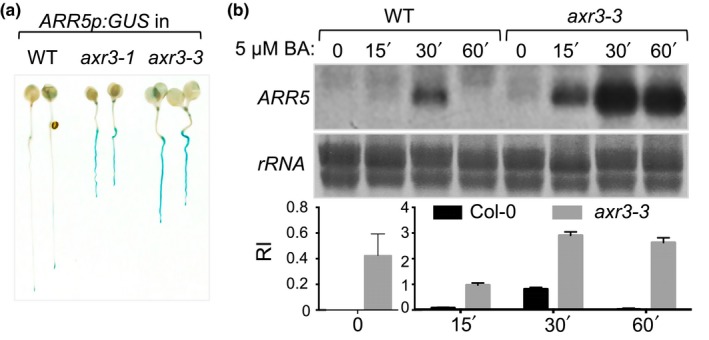
Auxin inhibits cytokinin signaling. (a) Expression pattern of *ARR5p:GUS* in 5‐day‐old Col‐0 (WT), *axr3‐1*, and *axr3‐3* seedlings. (b) RNA gel blot analysis of the effect of treatment with the cytokinin BA on *ARR5* transcript accumulation in Col‐0 and *axr3‐3* seedlings. Plants were grown for 7 days and then treated with 5 μM BA for the denoted time interval. Methylene blue‐stained membrane region with ribosomal RNAs (*rRNA*) is shown as a loading control. Relative intensity (RI) of the *ARR5* signal is shown below the loading control and is presented as mean ± *SD* of two biological replicates. Two different *y* axes are shown to allow the visualization of the difference in the untreated controls

An effective way to analyze the cytokinin response in tissues and plant organs is by using the *ARR5p:GUS* transgenic reporter line in which the promoter of the primary cytokinin response gene *ARR5* is fused to the coding region of β‐glucuronidase (D'Agostino, Deruere, & Kieber, 2000). To test how auxin affects the cytokinin transcriptional response in whole plants, we introgressed the *ARR5p:GUS* transgene into the auxin‐resistant mutants *axr2‐1, axr3‐1*, and *axr3‐3* that carry stabilization mutations in the AUX/IAA response repressors IAA7 and IAA17, respectively (Gray, Kepinski, Rouse, Leyser, & Estelle, 2001; Nagpal et al., 2000; Rouse, Mackay, Stirnberg, Estelle, & Leyser, 1998). We found that the *ARR5p:GUS* expression levels were higher in all tested *axr* mutants compared to wild‐type plants expressing the same reporter gene and the increased accumulation of blue‐colored chloro‐bromoindigo was observed not only in roots but also in hypocotyls, cotyledons, and flowers of *axr* seedlings (Figure 1a, Supporting Information Figure S1a,b). The increase in the *ARR5p:GUS* expression in *axr2* was not as strong as in the *axr3* mutant background and we hypothesized that this difference in reporter expression level reflects the strength of the auxin resistance phenotype (Supporting Information Figure S1a). To test this, we introgressed the *DR5p:GUS* transgene, which is induced by auxin and widely used to monitor the primary auxin response at the transcriptional level (Sabatini et al., 1999), into the *axr2‐1* and *axr3‐3* backgrounds. Treatment with 500 nM NAA for 4 hr was sufficient to induce *DR5p:GUS* expression in the wild type (Supporting Information Figure S1c). The *DR5p:GUS* expression was also induced in the *axr2‐1* mutant, albeit at lower levels compared to the wild type and was nearly undetectable in the *axr3‐3* mutant, thus confirming that *axr3* mutants have a stronger defect in auxin signaling than *axr2* (Supporting Information S1c). These results suggested that endogenous auxin (i.e., the steady‐state active auxin in plants not treated with exogenous hormones) inhibits the response to endogenous cytokinin (the steady‐state active cytokinin in untreated plants) and that auxin resistance relieves this inhibition.

Next, we compared the steady‐state levels and cytokinin induction kinetics of the *ARR5* transcript in the wild‐type and the *axr3‐3* mutant plants. The expression of type‐A *ARR* genes is characterized by low steady‐state levels, a fast cytokinin‐induced accumulation of transcripts, and finally, a decline caused by the accumulation of type‐A ARR response inhibitor proteins (D'Agostino et al., 2000). The *ARR5* transcript steady‐state level and its induction by treatment with the cytokinin 6‐benzyladenine (BA) were higher and the duration of its induced state was longer in the *axr3‐3* mutant compared to the wild type (Figure 1b). Thus, auxin‐resistant *axr3* seedlings are hypersensitive to cytokinin and have an increased cytokinin response capacity.

We next tested if auxin also inhibits cytokinin signaling at higher cytokinin concentrations. A dose–response analysis revealed that the *ARR5p:GUS* transgene is induced by BA at a concentration as low as 25 nM (Supporting Information Figure S2a). Cotreatment with the auxin 1‐naphtaleneacetic acid (NAA) suppressed the *ARR5p:GUS* induction caused by 50 nM BA (Supporting Information Figure S2b). However, this suppression required a high concentration of NAA (5 μM) and was localized to the upper region of the root (Supporting Information Figure S2b). We observed no NAA‐induced suppression of the *ARR5p:GUS* expression in seedlings treated with higher BA concentrations (e.g., 200 nM; Supporting Information Figure S2c). We concluded that auxin inhibits the response to low concentrations of cytokinin but is ineffective if the cytokinin content is high (e.g., as a consequence of the BA treatment).

### 3.2 Cytokinin insensitivity suppresses cytokinin‐related phenotypes of *axr3*

**Figure 2:**
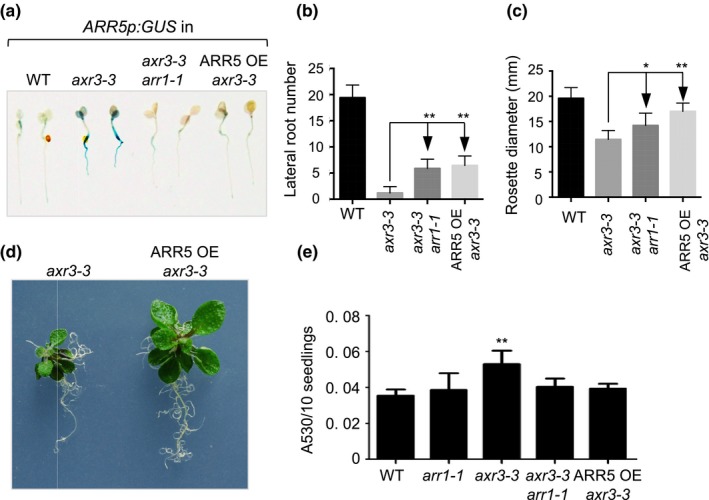
Cytokinin‐related phenotypes of the *axr3‐3* mutant. (a) Expression patterns of *ARR5p:GUS* in 4‐day‐old Col‐0 (WT), *axr3‐3, axr3‐3 arr1‐1*, and *35Sp:ARR5 axr3‐3* (ARR5 OE) seedlings. (b) Number of lateral roots emerging from the primary root of plants grown on vertically positioned plates for 18 days. The results are presented as mean ± *SD* (*n* ≥ 10). **p ≤ 0.01 compared to *axr3‐3* (one‐way ANOVA with Bonferroni's multiple comparisons test). (c) Rosette diameter of 18‐day‐old plants grown on horizontal plates. The results are presented as mean ± *SD* (*n* ≥ 10). *p ≤ 0.05 and **p ≤ 0.01 compared to *axr3‐3* (one‐way ANOVA with Bonferroni's multiple comparisons test). (d) Eighteen‐day‐old *axr3‐3* and ARR5 OE *axr3‐3* plants are shown to illustrate the differences in rosette sizes and root branching. (e) Relative anthocyanin content in 12‐day‐old plants. Pools of 10 plants (three biological replicates per line) were used for extraction and the results are presented as mean ± *SD*. **p ≤ 0.01 compared to Col‐0 (one‐way ANOVA with Bonferroni's multiple comparisons test)

To test if the enhanced cytokinin signaling in *axr3* seedlings contributes to the *axr3* developmental phenotype, we introduced two cytokinin‐resistant loci into the *axr3‐3* background. The first locus was the *arr1‐1* mutation which leads to decreased cytokinin sensitivity as it inactivates ARR1, a key member of the type‐B ARR family member of cytokinin response activators (Sakai et al., 2001). The second locus was the transgene *35Sp:ARR5* that overexpresses the cytokinin response inhibitor ARR5 (Li et al., 2013). Both loci suppressed the *GUS* expression in *ARR5p:GUS axr3‐3* plants providing further proof that the increased expression of the primary cytokinin response gene *ARR5* in *axr3* plants is caused by enhanced cytokinin signaling (Figure 2a).

Next, we analyzed the effects of *arr1‐1* and *35Sp:ARR5* on development and found that these cytokinin resistance loci suppress several *axr3‐3* phenotypes that could be caused by increased cytokinin action. First, we found that the strong reduction in the number of lateral roots of *axr3‐3* was partially alleviated in both *arr1‐1 axr3‐3* and *ARR5p:GUS axr3‐3* lines (Figure 2b). This suggested that the low lateral root number in *axr3‐3* seedlings is caused by a combined decrease in sensitivity to auxin, which functions as a promoter of lateral root formation (Du & Scheres, 2018), and increased action of cytokinin, which is a known inhibitor of lateral root formation (Laplaze et al., 2007; Riefler, Novak, Strnad, & Schmulling, 2006). The next two cytokinin‐related *axr3‐3* phenotypes were observed in shoots. First, we found that *axr3‐3* rosette leaf expansion was increased by the *arr1‐1* and *35Sp:ARR5* loci (Figure 2c,d). Although cytokinin is a plant growth promoter, it is well known that cytokinin treatments and transgenes that cause constitutive cytokinin signaling lead to a reduction in leaf expansion (Kurepa, Li, Perry, & Smalle, 2014; To et al., 2004). Therefore, increased cytokinin signaling in *axr3* seedlings is expected to cause reduced rosette growth—which is indeed the case—and this can be alleviated by introducing cytokinin resistance loci (Kurepa et al., 2014; To et al., 2004). Finally, we also found that *axr3‐3* seedlings have increased anthocyanin content, a phenotype that is also associated with increased cytokinin action (Deikman & Hammer, 1995). Both *arr1‐1* and *35Sp:ARR5* suppressed this cytokinin‐related phenotype (Figure 2e). We concluded that enhanced cytokinin signaling in *axr3* mutants underlies some of the *axr3* shoot and root phenotypes.

### 3.3 ARF7 inhibits cytokinin signaling

**Figure 3:**
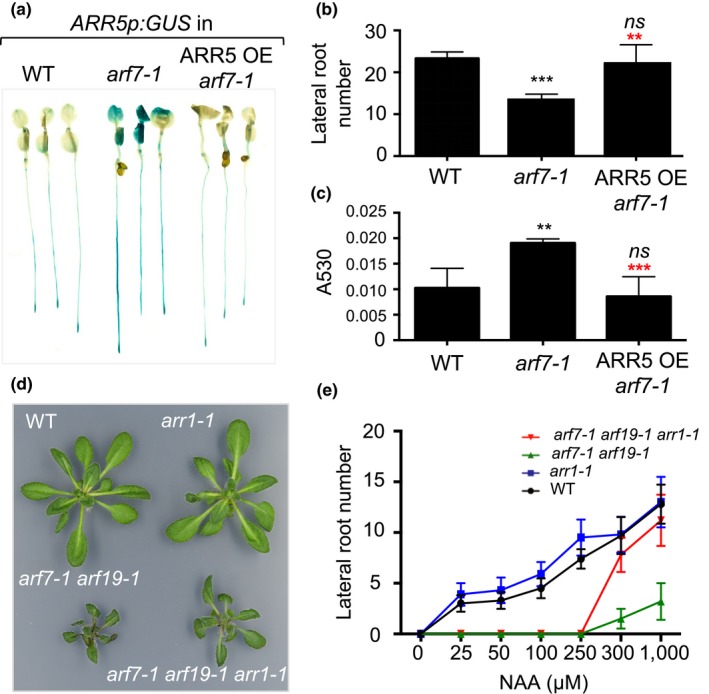
ARF7 inhibits cytokinin signaling. (a) Expression analyses of *ARR5p:GUS* in 5‐day‐old Col‐0 (WT), *arf7‐1*, and the *35Sp:ARR5 arf7‐1* (ARR5 OE) seedlings. (b) Lateral root number on the main root of 18‐day‐old plants grown on vertical plates. The results are presented as mean ± *SD* (*n* ≥ 10). ns, not significant; ***p ≤ 0.001 compared to WT, ** (red), *p* ≤ 0.01 compared to *arf7‐1* (one‐way ANOVA with Bonferroni's multiple comparisons test). (c) Relative anthocyanin content in 12‐day‐old seedlings. Pools of 10 plants were used for extraction and the results are presented as mean absorption at 530 nm per 10 seedlings (A530) ± *SD* (*n* = 3). ns, not significant; **p ≤ 0.01 compared to WT, *** (red), *p* ≤ 0.01 compared to *arf7‐1* (one‐way ANOVA with Bonferroni's multiple comparisons test). (d) Representative 3‐week‐old plants illustrating the rosette size difference between the *arf7‐1 arf19‐1* and *arf7‐1 arf19‐1 arr1‐1* lines. (e) Root induction frequencies from root explants of 6‐day‐old seedlings incubated for 11 days on the denoted concentrations of NAA. The results are presented as mean ± *SD* (*n* ≥ 10)

AXR3 inhibits the auxin response by inhibiting ARFs, which are transcriptional regulators of primary auxin response genes. Similar to the *axr3* mutants, the *ARR5p:GUS* reporter transgene was expressed at a higher level in the auxin‐insensitive *arf7‐1* mutant (Figure 3a) and was induced more strongly in response to cytokinin treatment (Supporting Information Figure S3). We then transformed *ARR5p:GUS arf7‐1* lines with the *35Sp:ARR5* transgene that causes cytokinin resistance (Supporting Information Figure S4). The expression of *ARR5p:GUS* was reduced in the *35Sp:ARR5 ARR5p:GUS arf7‐1* plants compared to the *ARR5p:GUS arf7‐1* plants and was similar to that detected in *ARR5p:GUS* expressed in the wild type (Figure 3a). Similar to *axr3* mutants, *arf7* mutants are characterized by decreased lateral root formation (Overvoorde et al., 2005; Wilmoth et al., 2005) and this phenotype was also reversed by the *35Sp:ARR5* transgene (Figure 3b). Anthocyanin accumulation, which is upregulated in *arf7‐1*, was also reverted to wild‐type levels in *35Sp:ARR5 arf7‐1* plants (Figure 3c). These results confirm the conclusion we reached with the *axr3* analyses: auxin is an inhibitor of endogenous cytokinin signaling (i.e., the cytokinin signaling in untreated plants) and this effect is mediated by the canonical auxin response pathway.

Compared to *arf7‐1*, the *arf7‐1 arf19‐1* double mutant is characterized by a stronger auxin resistance, a near complete absence of lateral root formation and a semidwarf rosette phenotype (Overvoorde et al., 2005; Wilmoth et al., 2005). Introgression of *arr1‐1* into *arf7‐1 arf19‐1* partially suppressed the semidwarf rosette phenotype of the double mutant indicating that enhanced cytokinin action also plays a role in this developmental change (Figure 3d). However, the *arr1‐1* mutation did not suppress the lateral root phenotype of *arf7‐1 arf19‐1* seedlings. We hypothesized that lateral root formation in *arf7‐1 arf19‐1* is halted because the auxin action is below a critical threshold level and lateral root growth cannot be initiated by introduction of the *arr1‐1* mutation. If true, supplementing the growth media with a high enough auxin concentration could activate the developmental program and the effect of the cytokinin insensitivity in the *arr1‐1 arf7‐1 arf19‐1* triple mutant could be unmasked. To test that, we analyzed the lateral root formation in an NAA dose–response assay on root explants (Figure 3e). Indeed, at higher NAA doses, the number of lateral roots reached ~90% of the wild‐type levels in *arr1‐1 arf7‐1 arf19‐1* root explants and only ~10% in *arf7‐1 arf19‐1*.

### 3.4 Cytokinin inhibits auxin signaling

**Figure 4:**
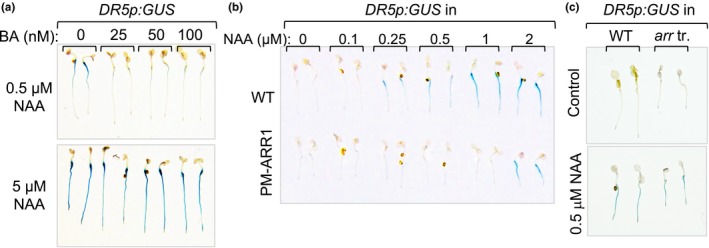
Cytokinin inhibits auxin signaling. (a) Expression patterns of *DR5p:GUS* in 5‐day‐old Col‐0 seedlings cotreated for 4 hr with the denoted combinations of NAA and BA. (b) Expression patterns of *DR5p:GUS* in 4‐day‐old Col‐0 (WT) and phosphomimic *35Sp:ARR1* ^*D94E*^ (PM‐ARR1) seedlings treated with NAA. The length of the GUS reaction was timed to reveal the *DR5p:GUS* expression differences between the WT and *35Sp:ARR1* ^*D94E*^ backgrounds. (c) Expression patterns of *DR5p:GUS* in 5‐day‐old WT and *arr1‐3 arr10‐5 arr12‐1* seedlings treated for 4 hr with 0.5 μM of NAA

Finally, we used the *DR5p:GUS* reporter line to test whether this auxin–cytokinin signaling interaction is uni‐ or bidirectional (Sabatini et al., 1999). Auxin‐treated *DR5p:GUS* plants were cotreated with a range of BA doses starting with the lowest dose previously determined to induce the expression of *ARR5p:GUS* (Figure 4a and Supporting Information Figure S2a). The lowest BA dose (25 nM) was sufficient to suppress the expression of *DR5p:GUS* induced with 500 nM NAA and the higher BA doses did not substantially add to this effect (Figure 4a). Similar to the effect of NAA on *ARR5p:GUS* expression (Supporting Information Figure S2c), which illustrated that the auxin suppression could be overcome by simply increasing the cytokinin dose, the BA‐mediated suppression of auxin‐inducible *DR5p:GUS* expression was abolished by increasing the auxin dose 10‐fold (Figure 4a).

To independently confirm a role of cytokinin signaling in the suppression of auxin signaling, we introgressed the *DR5p:GUS* transgene into the phosphomimic *35Sp:ARR1* ^*D94E*^ line which has a constitutive cytokinin response (Kurepa et al., 2014). Treatment of *35Sp:ARR1* ^*D94E*^ *DR5p:GUS* with a range of NAA doses showed that constitutive cytokinin signaling indeed suppresses the *DR5p:GUS* expression at lower NAA doses but becomes ineffective as the NAA dose increases (Figure 4b). Next, we tested if the opposite holds true, whether cytokinin resistance increases auxin signaling. Introgression of the *DR5p:GUS* transgene into the strong cytokinin‐resistant triple mutant *arr1‐3 arr10‐5 arr12‐1* revealed no increase in auxin signaling in untreated plants, and we also did not observe an enhanced induction of *DR5p:GUS* expression in response to treatments with auxin (Figure 4c). As the cytokinin induction of *ARR5p:GUS* was enhanced in auxin‐resistant mutants (Supporting Information Figure S3) and the auxin induction of *DR5p:GUS* was not altered in the cytokinin‐resistant seedlings, we concluded that the cytokinin at levels present in nontreated plants does not impact auxin signaling and that the signaling relation between auxin and cytokinin is unidirectional.

## 4 DISCUSSION

In this study, we have shown that the plant hormones auxin and cytokinin exert bidirectional inhibitory control over each other's signaling pathways in root and shoot organs, thus outside of apical meristems. These mutual inhibitory controls are asymmetrical and allow both antagonistic and additive hormone action. Cytokinin and auxin act antagonistically at low to medium concentrations of both hormones, and only at high concentrations, they act additively. The increased cytokinin signaling in auxin‐resistant mutants implies that, at the levels of both hormones in untreated plants, cytokinin signaling is suppressed by auxin action in most parts of the plant. However, a modest increase in cytokinin is sufficient to overcome this inhibition and this is accompanied by the simultaneous inhibition of auxin signaling. This antagonistic relationship ends when both hormone concentrations are high and both responses are uninhibited.

Here, we present evidence that inhibition of cytokinin signaling by auxin contributes to the control of both root and shoot growth. The discovery that the introduction of cytokinin resistance loci partially suppressed some of the auxin‐resistant mutant phenotypes (e.g., the reduced leaf expansion, increased anthocyanin accumulation, and decreased lateral root formation) suggests that the actions of auxin on root and shoot growth involve suppression of cytokinin signaling to facilitate auxin‐promoted processes.

Although the antagonistic action of auxin and cytokinin was unidirectional in untreated plants grown under laboratory conditions (auxin inhibits cytokinin action, but cytokinin does not inhibit auxin action), it is likely that the cytokinin inhibition of auxin signaling, which was observed in response to treatment with exogenous BA, also plays a role in plant root and shoot development. Several environmental conditions, for example, are known to temporarily increase cytokinin content either by enhancing cytokinin biosynthesis or by decreasing cytokinin degradation and inactivation (Bielach, Hrtyan, & Tognetti, 2017; Hirose et al., 2008). Although, one can envision that, under these conditions, cytokinin action impacts auxin signaling and auxin‐regulated development, additional studies are needed to show if this exogenous, pharmacological suppression has an internal functional equivalent.

Our results show that decreased auxin sensitivity enhances cytokinin signaling in all of the tested organs (i.e., roots, shoots, and reproductive tissues) indicating that these two signaling pathways cannot be disconnected. This raises an intriguing possibility that the antagonistic interconnection of auxin and cytokinin reflects an interaction at the signaling level. Such an interrelation of signaling pathways may serve as a basic regulatory framework which is then modified by developmentally and environmentally controlled changes in the biosynthesis, metabolism, and transport of both hormones, the expression of genes that encode the respective signaling pathway components and finally, on the developmental context. Consequently, it is possible that the mechanism that governs this auxin–cytokinin inhibition does not involve networks that were previously identified in specific tissues and zones such as meristems and vasculature. For example, the auxin‐inducible cytokinin response inhibitor AHP6 is important for root apical meristem development, root vascular patterning, and robustness of phyllotaxy at the shoot apical meristem (Besnard et al., 2014; Bishopp et al., 2011; Mähönen et al., 2006). However, loss of AHP6 function enhances cytokinin action only in these specific tissues and cell types and leads to modest changes in overall plant development which is consistent with the cell type‐specific expression pattern of the *AHP6* gene (Besnard et al., 2014; Bishopp et al., 2011; Mähönen et al., 2006). The *ahp6* mutants do not accumulate anthocyanins, their leaf expansion rates do not differ from the wild type, and although they have a mild decrease in lateral root emergence, their overall lateral root density was not significantly different from wild‐type plants (Moreira, Bishopp, Carvalho, & Campilho, 2013). In contrast, increased anthocyanin content, decreased leaf expansion, and decreased lateral root formation are all phenotypes of strong auxin‐resistant mutants that have a plant‐wide increase in cytokinin signaling. To conclude, we hypothesize that in addition to feedback mechanisms that regulate the strength of the response of each hormone separately and mechanisms that coregulate auxin and cytokinin action in specific tissues such as apical meristems, plants have an additional system that mediates auxin and cytokinin signaling interactions at the organismal level.

Finally, it needs to be noted that since we used only signaling mutants and transgenic lines and not biosynthesis mutants, our conclusions about the effects of both hormones on each other's signaling pathways are partially circumstantial. Analyses of biosynthesis mutants are expected to reveal a higher level of complexity of the effects of hormone interactions on growth, if nothing else than because of the known reciprocal controls that these two hormones have on each other's accumulation (Jones et al., 2010; Schaller et al., 2015). It is to be expected that many additional facets of interaction between the control mechanisms of hormone levels and the regulation of signal transduction mechanisms will be uncovered and these interactions are likely to include multiple feedback mechanisms.

## AUTHOR CONTRIBUTIONS

J.K., T.E.S, and J.S conducted the experiments. J.K. and J.S designed the experiments and wrote the paper. All authors read and approved the final manuscript.
