# Supplementary material for: Antagonistic activity of auxin and cytokinin in shoot and root organs
Source: Plant Direct. 2019 Feb 25;3(2):e00121. doi: 10.1002/pld3.121 (PMC6508789; doi:10.1002/pld3.121)
Supplement: Supplementary file 1 [file PLD3-3-e00121-s001.pdf]

Supporting Information

Figure S1. Increased *ARR5p:GUS* expression in *axr* mutants is correlated with the strength of the auxin resistance.

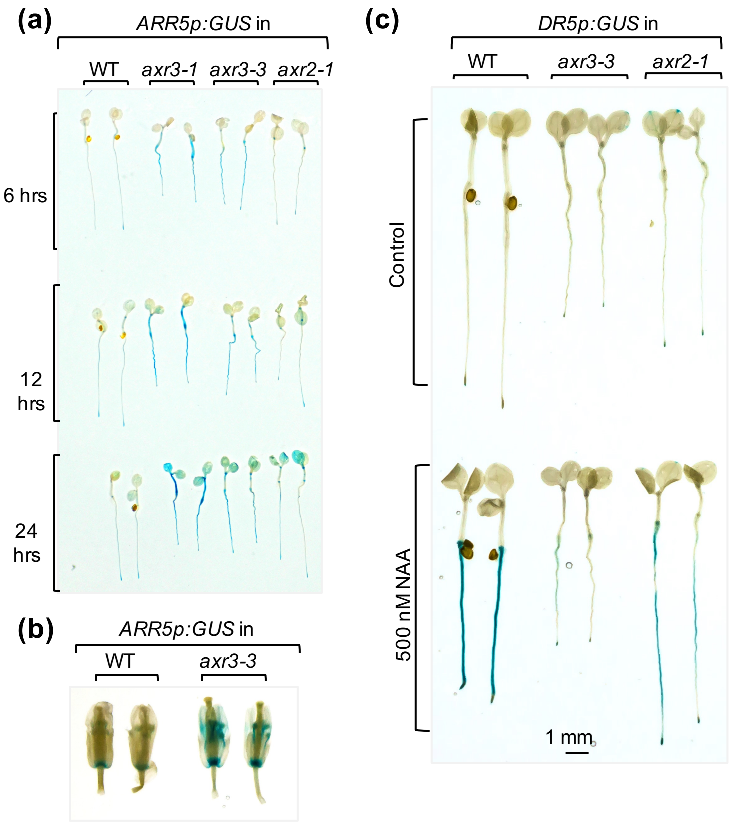

(a) *ARR5p:GUS* expression in 5-day-old seedlings of the Col-0 wild type (WT) and AUX/IAA mutants *axr3-1, axr3-3* and *axr2-1.* The GUS reaction was stopped at the denoted time points to visualize the different expression levels between *axr3* and *axr2* mutants (at the 6 and 12 hrs. time points) and between *axr2* seedlings and the wild type (at the 24 hrs. time point).

(b) *ARR5p:GUS* expression in flowers of the wild type and the *axr3-3* mutant both containing the *ARR5::GUS* transgene.

(c) *DR5p:GUS* expression in 5-day-old wild type, *axr3-3* and *axr2-1* plants treated for 4 hours with 500 nM of the auxin NAA. The length of the GUS reaction was timed to reveal the *DR5p:GUS* expression differences between the *axr* mutant backgrounds.

Co-treatment with high auxin concentration suppresses cytokinin effects

To determine the minimal effective dose of BA for the induction of the *ARR5p:GUS* reporter gene, we treated the *ARR5p:GUS* line for 4 hours with a range of BA doses (Figure S2(a)). After a 2-hour long incubation with the GUS substrate X-gluc, we determined that a minimum of 25 nM BA is needed for a visible accumulation of the blue colored chloro-bromoindigo in roots of 4-day-old seedlings. Co-treatments with 50 nM BA and different doses of the auxin 1-naphtaleneacetic acid (NAA) revealed that the expression of *ARR5p:GUS* is suppressed in the upper part of the root at the high NAA concentration of 5 μM (Figure S2(b)). However, co-treatments with higher BA concentrations (e.g., 200 nM, Figure S2(c)) reversed the NAA-dependent inhibition of *ARR5p:GUS* expression. Because the auxin inhibition of cytokinin-induced *ARR5p:GUS* involved a high concentration of auxin that is likely not to be representative of how the endogenous auxin/cytokinin interaction is regulated, most of our experiments focused on the analyses of auxin resistant mutants that allow to determine the effects of endogenous auxin.

Figure S2. High auxin dose suppresses the induction of *ARR5p:GUS* by a low BA dose.

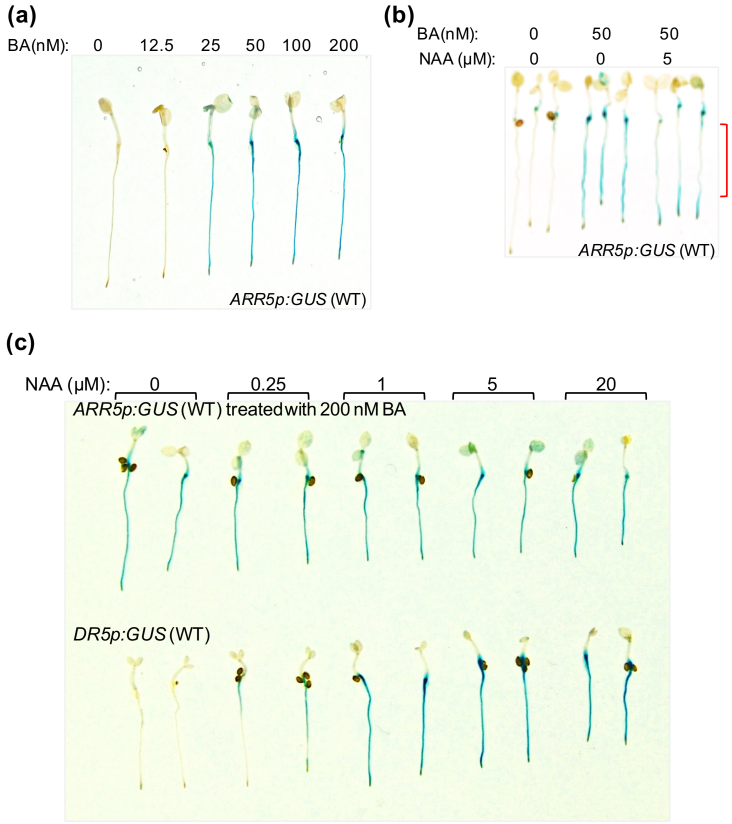

Figure S2. High auxin dose suppresses the induction of *ARR5p:GUS* by a low BA dose.

(a) GUS expression in 5-day-old *ARR5p:GUS* transgenic seedlings treated for 4 hours with a concentration range of the cytokinin BA.

(b) GUS expression in 5-day-old *ARR5p:GUS* transgenic seedlings treated for 4 hours with the denoted concentrations of BA and NAA.

(c) GUS expression in 5-day-old 200 nM BA-treated *ARR5p:GUS* co-treated with a range of NAA concentrations for 4 hours. *DR5p:GUS* transgenic seedlings treated with the same NAA concentration range are shown as a control for the auxin effect.

Figure S3. BA-induction of *ARR5p:GUS* is enhanced in auxin resistant mutants.

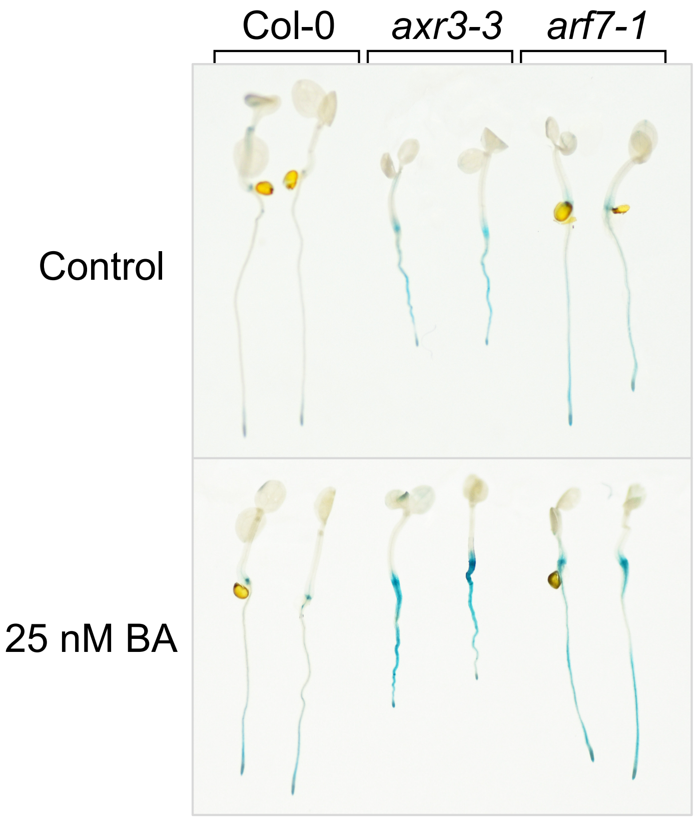

ARR5p:GUS expression in 5-day-old seedlings of the wild type Col-0 and auxin resistant mutants *axr3-3* and *arf7-1* treated for 4 hours with 25 nM of the cytokinin BA. The length of the GUS reaction was timed to reveal the *ARR5p:GUS* expression differences between the wild type, *axr3* and *arf7* mutant backgrounds.

Figure S4. Cytokinin resistance of the *35Sp:ARR5 ARR5p:GUS arf7-1* line.

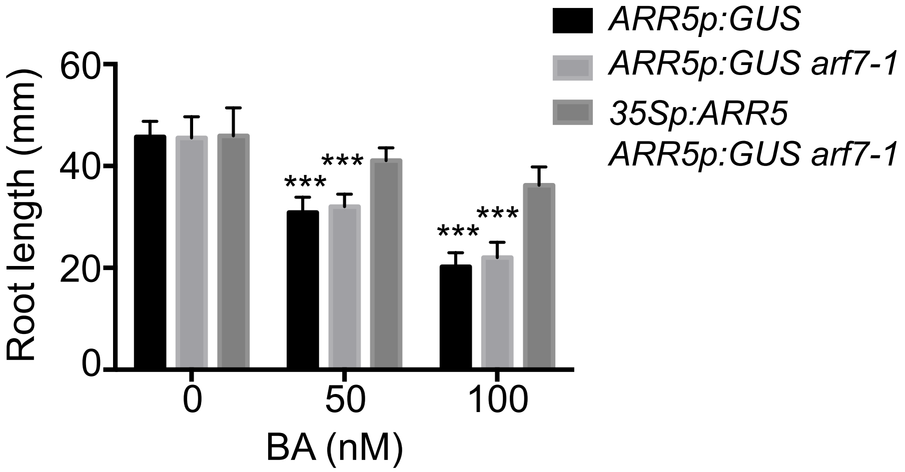

Seedlings of the denoted lines were grown for 4 days on horizontal plates and then transferred to vertical plates with the denoted BA concentration range. Root lengths were measured after an additional 8 days of growth.

Table S1. List of mutants and transgenic lines used in this study.

The genetic background of the mutant or transgenic line is noted in the parenthesis. Type referest to: P/I, point or irradiation mutations; IM, insertion mutations; TL, *Agrobacterium tumefactions* transformation generated transgenic lines. If the line carries a selectable marker, its name is listed in the column “selection”. If a visible phenotype is used for selection, it is listed under “Phenotype”. Km, kanamycin; Basta, phosphinothricin; Hyg, hygromycin.

Name	Type	Selection	Phenotype	Ref
axr2-1	P/I		Auxin resistance	(*1*)
ARR5p:GUS	TL	Km		(*2*)
ARR5p:GUS axr2-1	TL, P/I	Km	Auxin resistance	this study
axr3-1	P/I		Auxin resistance	(*3*)
ARR5p:GUS axr3-1	TL, P/I	Km	Auxin resistance	this study
axr3-3	P/I		Auxin resistance	(*3*)
ARR5p:GUS axr3-3	TL, P/I	Km	Auxin resistance	this study
35Sp:ARR1^{D94E}	TL	Basta	Constitutive cytokinin response	(*4*)
ARR5p:GUS 35Sp:ARR1^{D94E}	TL	Km<br>Basta	Constitutive cytokinin response	this study
arr1-1	IM	Hyg	Cytokinin resistance	(*5*)
axr3-3 arr1-1	P/I<br>IM	Hyg	Auxin resistance<br>Cytokinin resistance	this study
ARR5p:GUS axr3-3 arr1-1	P/I<br>IM	Hyg<br>Km	Auxin resistance<br>Cytokinin resistance	this study
35Sp:ARR5	TL	Basta	Cytokinin resistance	(*6*)
ARR5p:GUS 35Sp:ARR5 axr3-3	TL	Km<br>Basta	Cytokinin resistance<br>Auxin resistance	this study
arf7-1	IM		Auxin resistance	(*7*)
ARR5p:GUS arf7-1	TL<br>IM		Auxin resistance	this study
ARR5p:GUS 35Sp:ARR5 arf7-1	TL<br>IM	Basta	Cytokinin resistance<br>Auxin resistance	this study
arf7-1 arf19-1	IM		Auxin resistance	(*7*)

arr1-1 arf7-1 arf19-1	IM	Hyg	Cytokinin resistance<br>Auxin resistance	this study
DR5p:GUS	TL	Km		(*8*)
DR5p:GUS 35Sp:ARR1^{D94E}	TL	Km<br>Basta	Constitutive cytokinin response	this study
arr1-3 arr10-5 arr12-1	IM		Cytokinin resistance	(*9*)
DR5p:GUS arr1-3 arr10-5 arr12-1	TL<br>IM		Cytokinin resistance	this study

1. A. K. Wilson, F. B. Pickett, J. C. Turner, M. Estelle, A dominant mutation in Arabidopsis confers resistance to auxin, ethylene and abscisic acid. *Mol Gen Genet* **222**, 377-383 (1990).
2. I. B. D'Agostino, J. Deruere, J. J. Kieber, Characterization of the response of the Arabidopsis response regulator gene family to cytokinin. *Plant Physiol* **124**, 1706-1717 (2000).
3. D. Rouse, P. Mackay, P. Stirnberg, M. Estelle, O. Leyser, Changes in auxin response from mutations in an AUX/IAA gene. *Science* **279**, 1371-1373 (1998).
4. J. Kurepa, Y. Li, S. E. Perry, J. A. Smalle, Ectopic expression of the phosphomimic mutant version of Arabidopsis response regulator 1 promotes a constitutive cytokinin response phenotype. *BMC Plant Biol* **14**, 28 (2014).
5. H. Sakai *et al.*, ARR1, a transcription factor for genes immediately responsive to cytokinins. *Science* **294**, 1519-1521 (2001).
6. Y. Li, J. Kurepa, J. Smalle, AXR1 promotes the Arabidopsis cytokinin response by facilitating ARR5 proteolysis. *Plant J* **74**, 13-24 (2013).
7. Y. Okushima *et al.*, Functional genomic analysis of the AUXIN RESPONSE FACTOR gene family members in Arabidopsis thaliana: unique and overlapping functions of ARF7 and ARF19. *Plant Cell* **17**, 444-463 (2005).
8. S. Sabatini *et al.*, An auxin-dependent distal organizer of pattern and polarity in the Arabidopsis root. *Cell* **99**, 463-472 (1999).
9. R. D. Argyros *et al.*, Type B response regulators of Arabidopsis play key roles in cytokinin signaling and plant development. *Plant Cell* **20**, 2102-2116 (2008).
