# Supplementary material for: Antagonistic activity of auxin and cytokinin in shoot and root organs
Source: Plant Direct. 2019 Feb 25;3(2):e00121. doi: 10.1002/pld3.121 (PMC6508789; doi:10.1002/pld3.121)
Supplement: Supplementary file 2 [file PLD3-3-e00121-s002.pdf]

Responses

We have responded to all comments point by point and specified the edits in the new version of the manuscript.

Reviewers' Comments

Reviewer #1:

I am satisfied with the way the authors addressed my concerns. I think this manuscript is now suitable for publication.

Thank you.

Reviewer #3:

In the article Antagonistic activity of auxin and cytokinin in shoot and root organs by Jasmina Kurepa, Timothy E. Shull, and Jan A. Smalle, new aspects of the crosstalk between auxin-cytokinin are examined. Dissecting the different layers of interaction between these two hormones has been proven to be challenging. The results in this manuscript contribute to gain a better understanding of specific features of this interaction beyond the well-studied role of auxin/cytokinin and their interplay in meristematic regions.

Comment #1

On the new text that have been edited, it can be read: "Lateral organ formation in roots is essentially different in that..."

The authors are partially correct on this statement. The formation of the meristem of the emerging lateral root might be independent from the apical meristem. However, the location of the lateral root and the triggering signals are determined by the priming caused by the apical meristem. This sentence should be accordingly modified to include this concept. Periodic Lateral Root Priming: What Makes It Tick? The Plant Cell, 2017 by Marta Laskowski and Kirsten H. ten Tusscher is an excellent review on the topic.

Response to comment #1

Thank you for pointing this out. We agree and we have modified the sentence in question and it now reads:

"Whether or not pericycle cells engage in the cell division needed to develop lateral root meristems depends on priming by the root apical meristem (*1*) and also on signaling mechanisms that reside outside of the root apical meristem."

Comment #2

I think the widespread use of the term "endogenous levels" might be misleading. Please, revise the use of this term across the manuscript.

For example, "These results suggested that endogenous auxin inhibits the response to endogenous cytokinin and that auxin resistance relieves this inhibition." The use of mutants and

treatments with exogenous NAA is not sufficient to state that the observed results are due to endogenous levels. 500 µM is much higher than the equivalent IAA physiological levels. In addition, some auxin signaling and transport mutants produce more IAA due to a compensatory effect which can be difficult to interpret.

Along these lines, on the sentence: "We concluded that auxin inhibits the response to low, endogenous concentrations of cytokinin but is ineffective if the cytokinin content is high" The text could be more concise by reflecting the fact that these are treatments with exogenous BA.

Response to comment #2

Part 1

We have used "endogenous levels" throughout the manuscript to describe the auxin and cytokinin levels that exist in plants that have not been treated with hormones. We were hoping that the use of the term endogenous would be understood from this perspective and that it would not invoke any speculations about the absolute amounts of the hormone or changes thereof.

However, the reviewer's comment has convinced us that there is a need for further clarification to avoid ambiguity. To that end, we have either:

1. removed the term (if the context was clear enough – e.g. "~~endogenous~~ *ARR5* transcript", p. 5),
2. specified the use of the term (e.g., "These results suggested that endogenous auxin (i.e., the steady-state active auxin in plants not treated with exogenous hormones) inhibits the response to endogenous cytokinin (the steady-state active cytokinin in untreated plants) and that auxin resistance relieves this inhibition.", p. 5),
3. or replaced it with for example "at levels present in non-treated plants" instead of "endogenous levels" (p. 10.)

Part 2

Our conclusion "These results suggested that endogenous auxin inhibits the response to endogenous cytokinin and that auxin resistance relieves this inhibition" is not based on results of hormone treatments, but rather on the observation that the untreated auxin resistant mutants *axr3* and *arf7* have increased expression of the primary cytokinin response gene *ARR5* and an overall increase in cytokinin-related developmental phenotypes consistent with increased cytokinin signaling (Figures 1a, 2a-e, 3a-c and S1b). This indicated that auxin resistance caused increased cytokinin action, which could be the result of increased cytokinin sensitivity to endogenous cytokinin in the absence of treatments and increased signaling capacity. This cytokinin hypersensitivity was subsequently confirmed by treatments with exogenous cytokinin, which served as an independent control but was not the experiment that yielded the original conclusion (Figures 1b and S3).

The reviewer correctly points out that some of our conclusions are based solely on hormone treatments (e.g., such as the inhibition of the response to exogenous auxin by exogenous BA). The results of treatments indeed do not provide conclusive evidence that this type of regulation also acts during the development of untreated plants that might have localized increases in

cytokinin content sufficient enough for counteracting and suppressing auxin signaling. We agree that this should be emphasized and have added the following part to the discussion section:

"Although, one can envision that under these conditions, cytokinin action impacts auxin signaling and auxin-regulated development, additional studies are needed to show if this exogenous, pharmacological suppression has an internal functional equivalent. ", p. 11

For the same reason, we have modified the sentence on p. 6
from:
"We concluded that auxin inhibits the response to low, endogenous concentrations of cytokinin but is ineffective if the cytokinin content is high"
to
"We concluded that auxin inhibits the response to low concentrations of cytokinin but is ineffective if the cytokinin content is high (e.g., as a consequence of BA treatment)."

Likewise, in the Discussion section on page 11, we have specified that inhibition of auxin signaling by cytokinin was the result of treatment with exogenous cytokinin. It now reads:

"Whereas the antagonistic action of auxin and cytokinin was unidirectional in untreated plants grown under laboratory conditions (auxin inhibits cytokinin action, but cytokinin does not inhibit auxin action), it is likely that the cytokinin inhibition of auxin signaling, which was observed in response to treatment with exogenous BA, also plays a role in plant root and shoot development."

Part 3
We have used 500 nM NAA (and not 500 µM NAA), a concentration used within the auxin research community for the testing of short-term molecular responses (as opposed to long-term growth responses which indeed involve lower concentrations of this hormone) (*2-6*).

Comment #3
In the discussion section: "Our results show that changes in auxin sensitivity influence cytokinin signaling throughout the plant, not just in specific tissues and developmental stages."

The impact of IAA on CKs signaling takes place in specific tissues and at specific developmental stages. Maybe, the auxin/CKs interaction described in this manuscript is not located in the same sites (tissue/organs) that have previously been characterized, but it is totally incorrect to state that signaling does not take place in specific tissues at specific developmental stages. Hormone signaling is by nature rather local/discrete than general/constitutive.

By the end of this paragraph, "plants have an additional system that regulates auxin and cytokinin signaling interactions at the organismal level." Maybe say at the "organ level". This sentence reflects an idea opposite to that in the previously mentioned phrase "not just in specific tissues and developmental stages". Kind of contradictory.

Response to comment #3

We agree with the reviewer that the sentence "Our results show that changes in auxin sensitivity influence cytokinin signaling throughout the plant, not just in specific tissues and developmental stages" was open to misinterpretation and needed to be further specified. We did not intend to say that the outcome of the auxin/cytokinin interaction uncovered here remains the same throughout the plant and does not lead to specific outcomes at the tissue and organ levels. Because we have found the same inhibitory relationship of auxin on cytokinin signaling in all of the tested tissues and organs, including roots, cotyledons, hypocotyls and flowers, we have to consider the possibility that these signaling pathways are connected regardless of cellular context. This does not mean that signaling is general or constitutive. To the contrary, such a signaling pathway would serve as a regulatory framework, the outcome of which would depend on developmentally and environmentally controlled changes in the biosynthesis, metabolism and transport of both hormones, in the expression levels of the genes that encode the respective signaling pathway components, and finally also on the developmental context. The additional and tissue-specific auxin/cytokinin signaling interactions observed previously in apical meristems and vascular systems would then represent additional layers of control that reflect the crucial importance of meristems for the accuracy of plant growth regulation.

We have modified the discussion section to reflect these specifications better and have added the following on page 11:

“Our results show that decreased auxin sensitivity enhances cytokinin signaling in all of the tested organs (i.e., roots, shoots and reproductive tissues) indicating that these two signaling pathways cannot be disconnected. This raises an intriguing possibility that the antagonistic interconnection of auxin and cytokinin reflects an interaction at the signaling level. Such an interrelation of signaling pathways may serve as a basic regulatory framework which is then modified by developmentally and environmentally controlled changes in the biosynthesis, metabolism and transport of both hormones, the expression of genes that encode the respective signaling pathway components and finally, on the developmental context.”

Comment #4

In the Experimental procedures section, for the hormonal treatments and also for the pictures in the figures showing the most representative seedlings for each treatment, could you please say how many times these experiments were repeated/ bio reps?

Response to comment #4

We have corrected this. The number of biological replicates and the number of technical replicates (e.g., seedlings per measurement) is specified in the Materials and Methods section and in the Figure Legends.

1. M. Laskowski, K. H. Ten Tusscher, Periodic Lateral Root Priming: What Makes It Tick? *Plant Cell* **29**, 432-444 (2017).
2. T. Prat *et al.*, WRKY23 is a component of the transcriptional network mediating auxin feedback on PIN polarity. *PLoS Genet* **14**, e1007177 (2018).
3. H. Li, Y. Cheng, A. Murphy, G. Hagen, T. J. Guilfoyle, Constitutive repression and activation of auxin signaling in Arabidopsis. *Plant Physiol* **149**, 1277-1288 (2009).
4. C. Weiste, W. Droge-Laser, The Arabidopsis transcription factor bZIP11 activates auxin-mediated transcription by recruiting the histone acetylation machinery. *Nat Commun* **5**, 3883 (2014).
5. Y. Hu, Q. Xie, N. H. Chua, The Arabidopsis auxin-inducible gene ARGOS controls lateral organ size. *Plant Cell* **15**, 1951-1961 (2003).
6. S. Wang, S. B. Tiwari, G. Hagen, T. J. Guilfoyle, AUXIN RESPONSE FACTOR7 restores the expression of auxin-responsive genes in mutant Arabidopsis leaf mesophyll protoplasts. *Plant Cell* **17**, 1979-1993 (2005).
